# Probabilistic Approaches to Better Quantifying the Results of Epidemiologic Studies

**DOI:** 10.3390/ijerph7041520

**Published:** 2010-04-01

**Authors:** Paul Gustafson, Lawrence C. McCandless

**Affiliations:** 1 Department of Statistics, University of British Columbia, 333-6356 Agricultural Road, Vancouver, B.C., V6T 1Z2, Canada; 2 Faculty of Health Sciences, Simon Fraser University, 8888 University Drive, Burnaby, B.C., V5A 1S6, Canada; E-Mail: lmccandl@sfu.ca

**Keywords:** confounding, epidemiologic methods, exposure misclassification, selection bias, sensitivity analysis

## Abstract

Typical statistical analysis of epidemiologic data captures uncertainty due to random sampling variation, but ignores more systematic sources of variation such as selection bias, measurement error, and unobserved confounding. Such sources are often only mentioned via qualitative caveats, perhaps under the heading of ‘study limitations.’ Recently, however, there has been considerable interest and advancement in probabilistic methodologies for more integrated statistical analysis. Such techniques hold the promise of replacing a confidence interval reflecting only random sampling variation with an interval reflecting all, or at least more, sources of uncertainty. We survey and appraise the recent literature in this area, giving some prominence to the use of Bayesian statistical methodology.

## 1. Introduction

Much of the methodological literature on inferring exposure-disease relationships from observational data looks, either implicitly or explicitly, at the best-case situation: a random sample from the study population can be obtained, and all pertinent variables can be measured without error on sampled individuals. Commensurately, in real applications it is common to see best-case quantitative methods applied in settings where the best-case assumptions likely, or perhaps surely, fail. This is often ameliorated with some qualitative discussion of how the best-case assumptions might be violated, and some speculation on what impact such violations may have had on the quantitative results which are reported.

Unfortunately, the alliance between the quantitative results based on best-case assumptions and the qualitative comments casting doubt on these assumptions is typically shaky. If the only quantitative results given pertain to the best-case analysis, then these results tend to form the take-away message of the research. However well-intended, caveats and provisos about the realism of best-case assumptions are easily set aside by readers. Plus, even the most diligent readers will not be able to turn the qualitative remarks into clear inferential summaries. For instance, say an estimate and 95% confidence interval are reported for an effect of interest, using a method which assumes best-case conditions. Then qualitative caveats are added about possible discrepancies between the actual sampling scheme and random sampling, possible imperfections in measuring the available variables, and omissions of variables which are possibly germane to the relationship of interest. There may be some intuition about the direction (if any) in which the estimate ought to be shifted given these concerns, but there is seldom clarity on how large a shift is needed. Worse still, there is no recipe to indicate how much wider the confidence interval ought to be in light of the caveats, and it can be hard to generate even rough intuition on this question. Quantitative results based on overly strong assumptions are just not readily synthesized with qualitative remarks on the possible violations of these assumptions.

A situation commonly faced by consumers of research is the best-case confidence interval for the effect of interest excludes the null value, but qualitative concerns are expressed about the likely divergence of reality from the best-case. Given such concerns, there is a literature on various forms of sensitivity analysis, going back to Cornfield’s work on tobacco smoking and lung cancer [1]. The basic idea is to consider a range of different *quantitative* assumptions about how the best-case assumptions could fail, and see what inference arises for the exposure-disease relationship under each assumption. Recently there has been emphasis on probabilistic forms of sensitivity analysis [2–8]. Instead of constructing a list of different assumptions about how best-case conditions are violated, a *probability distribution* is used to describe a spectrum of plausible assumptions.

Before proceeding with further discussion of probabilistic sensitivity analysis, we review some of the major ways in which best-case assumptions will often be violated in epidemiological investigations of exposure-disease relationships. We do this in the framework of exposure and disease variables which are labeled *X* and *Y* respectively, along with *p* possible confounding variables, denoted *C* = (*C*_1_, . . . ,*C_p_*).

### 1.1. Selection Bias

Implicit in a standard analysis is the assumption the data are representative of the study population. This is guaranteed for simple random sampling of (*Y*, *X*, *C*), but is more delicate for complex study designs. Cohort studies involve sampling on exposure, meaning that the prevalence of *X* is fixed by design. Individuals with rare exposures are more likely to be selected into the study. Provided that study participation does not depend on *Y* or *C*, then the investigator can study the distribution of the outcome within exposure groups at the end of follow-up.

In contrast, case-control studies involve sampling on the outcome, in the sense that the probability of being selected into the study will necessarily depend on *Y*. Particularly for rare diseases one can expect that members of the population with the disease will have a higher probability of participating in the study. The retrospective study design compares the exposure distribution amongst cases to that amongst controls, as a route to comparing the disease distribution amongst exposed to that amongst unexposed. Consequently, a crucial assumption is that the exposure distribution in the controls is representative of the general population.

Things go awry if the exposure affects the probability of selection. For example, in case-control studies of magnetic field exposure and childhood leukaemia, Mezei and Kheifets [9] speculate that higher socioeconomic status may reduce one’s probability of exposure but simultaneously increase the likelihood of participation as a control. In this case the summaries of the exposure within cases and controls are biased, making any comparisons less meaningful. In the epidemiology literature, this phenomenon is called *selection bias* and it has direct connections to the missing data literature in statistics. It is worth noting that randomized clinical trials can also be hampered by selection bias, if loss to follow-up is differential across study arms.

In concept selection bias is readily dealt with by modifying a standard analysis, *if it is known how inclusion probabilities depend on pertinent variables*. Particularly, methods for analyzing weighted data apply, with sampled individuals receiving less/more weight in the analysis if their inclusion probabilities are high/low. This directly adjusts for the selection bias, in a manner analogous to making ‘extra copies’ of those individuals who were sampled despite this being unlikely, while ‘destroying copies’ of those who were very likely to be sampled [10]. The problem, however, is that often selection bias is suspected, but without knowledge of how the inclusion probabilities depend on *X* and/or *Y* and/or *C*. At this point one must turn to sensitivity analysis.

### 1.2. Misclassification

Many variables of interest in epidemiologic studies are not easily measured. It may be expensive or even technically impossible to measure a particular variable without error. This could apply to an outcome variable, to an exposure variable, or to a confounding variable. There is a particular emphasis in the literature, however, on poor measurement of exposure. This arises in part because many human exposures of a toxicological or nutritional nature are inherently hard to measure well. It also arises in part because parameter estimates from statistical models explaining the conditional distribution of disease outcome given exposure are particularly susceptible to bias as a result of unacknowledged exposure measurement error.

In general, if enough is known about exposure measurement error then the effect of this error can be ‘undone’ statistically. Roughly speaking, this applies if the measured exposure *W* arises via a combination of the unobserved true exposure *X* and ‘random noise,’ where the distribution of this noise is known. Such knowledge does not suffice to recreate the actual *X* values, but it does allow valid estimation of the conditional association between *Y* and *X* given *C* from data on (*Y*, *W*, *C*), via suitable methods. Thus the bias can be undone. The same is not true of estimator variability, however. The estimator arising from suitable treatment of the (*Y*, *W*, *C*) data will be more variable than the standard estimator applied to (*Y*, *X*, *C*) data, were such data available.

Unfortunately, many epidemiological settings involve insufficient knowledge of the nature and distribution of exposure measurement error. Thus the problem devolves to one of sensitivity analysis. One can examine the inferences that arise about the desired exposure-disease relationship over a range of assumptions concerning the magnitude of exposure measurement error.

While not emphasized here, it should also be mentioned that poor measurement of confounding variables is also a challenging situation in practice. Intuitively, error in measuring confounders C should result in an analysis which is intermediate between confounder adjustment based on correctly measured C and a crude analysis without confounder adjustment. See Fewell *et al.* [11] for recent discussion of this problem.

### 1.3. Unobserved Confounding

A further challenge in epidemiology is unobserved confounding. Suppose that *U* = (*U*_1_, . . . , *U_q_*) denotes a collection of confounders that are unmeasured by happenstance or limitations in the data collection process. For example, the investigator may, out of necessity, use population databases or registries that contain only limited information on the determinants of the disease. Alternatively, one could imagine settings where there are *unknown* confounders in the sense that the investigator cannot identify all disease risk factors.

Suppose that we study the distribution of the outcome across levels of exposure, but without taking *U* into consideration. Then we will observe a mixing of the effect of the exposure (if any) with that of *U*. By definition, the components of *U* are unequally distributed between exposure groups. Thus any observed association between *X* and *Y* could be a spurious artifact of confounding.

The most intuitive approach to dealing with confounding–when the confounding variable is observed–is via stratified analysis. We estimate the exposure effect within levels of *U* and then pool the results if it is deemed appropriate. More generally, we can use model-based regression adjustment, standardization, or more advanced techniques involving propensity scores. Unfortunately, when *U* is missing none of these techniques can be used without strong assumptions about the magnitude of confounding by *U*.

In recent years the has been renewed interest in techniques for sensitivity analysis for unmeasured confounding; see Schneeweiss [12] or Greenland [13] for accessible reviews. A further refinement uses a probabilistic Bayesian approach that seeks more formal integration of the data with prior beliefs about the magnitude of bias. These methods allow the investigator to compute bias-corrected effect estimates that incorporate uncertainty from bias in addition to random error.

In what follows, Sections 2 through 4 illustrate sensitivity analysis applied to unobserved confounding, selection bias, and misclassification, respectively. Some concluding remarks are given in Section 5. The more technical arguments involved in Sections 2 and 4 appear in the Appendix.

## 2. Example: Acknowledging Unobserved Confounding in a Simple Setting

To give some simple illustrations of sensitivity analysis, we focus on a setting which doesn’t involve any observed confounding variables, and the only observed variables are the binary exposure *X* and the binary disease status *Y*. Moreover, these two variables are taken to be measured without error, and further it is assumed that there is no selection bias. Whether they arise from a cohort study or a case-control study, the observed data are typically reported in the form of a simple 2 × 2 table.

As an example, consider the data in Table 1, taken from MacMahon *et. al.* [14], and also emphasized in Jewell [15]. They concern a case-control study from the late 1970’s that identified an apparent association between coffee drinking and pancreatic cancer. The cases were 367 cancer patients from hospitals in Boston and Rhode Island. The 643 controls were selected from patients who were hospitalized by the same attending physicians that treated the cases. The exposure status is dichotomized according to whether or not a subject reports drinking at least one cup of coffee per day. Henceforth these data are referred to as the coffee and pancreatic cancer (CPC) data.

The crude odds ratio for these data is 2.75, which, assuming no selection bias or measurement error, we can regard as an estimate of the exposure-disease odds ratio in the study population. Standard methods give a 95% confidence interval around this estimate as (1.66, 4.55).

Now we focus on what inferences we might draw in the face of concern about unobserved confounding. To wit, suppose that we consider the simple case where a binary variable *U* is a potential confounder, but is not observed in the data. In fact, one could easily argue that there may be several missing confounders. However Wang and Krieger [16] have shown that the binary confounder case represents the worst-case scenario, and furthermore as we shall argue, it considerably simplifies articulating assumptions about bias. If *U* were observed, we could simply apply standard methods to infer the conditional association between *Y* and *X* given *U*. Particularly, a logistic regression model could be used to explain *Y* in terms of *X* and *U*.

Without the ability to observe *U*, sensitivity analysis can be implemented by constructing various scenarios regarding how *U* relates to the other variables, and then producing inference on the target quantity under each scenario. In the present setting, a scenario can be built upon specification of the following four quantities:

1. The prevalence of *U*, *i.e.*, is *U* = 1 rare or common? (More technically, the specification is made in terms of prevalence amongst unexposed controls.)
2. The extent to which *U* is associated with exposure *X*.
3. The extent to which *U* is associated with the disease indicator *Y*.
4. How the conditional association between *Y* and *X* given *U*, which is the target of inferential interest, depends on *U*.

For convenience, and in line with much of the literature on sensitivity analysis (see, for instance [3]), we deal with item 4 by assuming that the conditional odds ratio between *Y* and *X* given *U* = 0 is the same as the conditional odds ratio between *Y* and *X* given *U* = 1. This common odds ratio is then the single and unambiguous target of inference. Moreover, this target will differ from the unconditional odds ratio between *X* and *Y* if *U* is indeed a confounding variable. Note that assuming a common exposure-disease odds-ratio within strata defined by a confounder is the basis of pooled estimators such as the Mantel-Haenzel estimator, and corresponds to the assumption of no interaction between *X* and *U* if fitting a logistic regression model for *Y* in terms of *X* and *U*.

Items 1 through 3 are conveniently dealt with together by assuming a relationship of the form

(1) $$\text{logit}\text{Pr}(U=1|X,Y)=\alpha_{u}+\alpha_{xu}X+\alpha_{yu}Y.$$

Thus the parameter *α_u_* controls the prevalence of *U*, *i.e.*, expit(*α_u_*) is the prevalence of *U* = 1 amongst unexposed controls. Meanwhile, *α_xu_* and *α_yu_* control the associations between *X* and *U* and between *Y* and *U* respectively. More particularly, given the underlying properties of logistic regression models, exp(*α_xu_*) is the conditional odds ratio between *X* and *U* given *Y*, and similarly exp(*α_yu_*) is the conditional odds ratio between *Y* and *U* given *X*. Given the lack of an interaction term in the model described by equation (1), both these conditional odds ratios do not depend on the value of the variable being conditioned upon. To be clear, the parameters (*α_u_*, *α_xu_*, *α_yu_*) describe the (*U|X*, *Y*) relationship in the study population, but they cannot be estimated from the observed data because *U* is not observed. Given their role in determining how the crude association between *X* and *Y* is biased in estimating the target association between *X* and *Y* given *U*, and following Greenland [6], the components of *α* are referred to as *bias parameters*. More generally in sensitivity analysis we take bias parameters to refer to quantities governing the nature and extent of imperfection in the observed data, with these parameters being varied in the sensitivity analysis in order to acknowledge this imperfection.

As explained in further detail in the Appendix, via a log-linear model for the cell counts in the 2×2×2 table stratified by (*X*, *Y*, *U*), model (1) can be combined with a logistic regression model for (*Y*|*X*, *U*) of the form

(2) $$\text{logit}\text{Pr}(Y=1|X,U)=\beta_{y}+\beta_{xy}X+\alpha_{yu}U.$$

This model encapsulates the parameter of interest as exp(*β_xy_*), the conditional odds ratio between *X* and *Y* given *U* (for either *U* = 0 or *U* = 1). Note as well that the *U* coefficient in (2) must indeed be the same as the Y coefficient in (1), since both coefficients are the logarithm of the conditional odds-ratio between *U* and *Y* given *X*.

Thus a basic sensitivity analysis is comprised of assuming values for the three components of *α* and then estimating *β_xy_*. As outlined in the Appendix, this inference can be carried out via standard statistical software for fitting generalized linear models (and specifically log-linear models).

One point of interest under this set up is that if *U* is assumed to not be a confounder then inference about the conditional exposure-disease association given *U* will match exactly the inference about the unconditional exposure-disease association. Thus setting *α_xu_* = 0 *or* setting *α_yu_* = 0 will yield an estimate and confidence interval for exp(*β_xy_*) that match exactly the sample odds-ratio for (*X*, *Y*) and the associated confidence interval.

Another point of interest is that the labels *U* = 0 and *U* = 1 are arbitrary, and consequently the same inferences about *β_xy_* would arise if all three components of *α* were flipped in sign. One simple way to rule out this redundancy is to consider only non-positive values of *α_u_*. That is, without loss of generality we can assume that the prevalence of *U* = 1 amongst unexposed controls does not exceed 50%.

Returning to the CPC data given in Table 1, in Table 2 we give inferences for the target odds ratio exp(*β_xy_*), under different scenarios for the unobserved confounder *U*. We refer to this as a *tabular sensitivity analysis* (TSA), though others might prefer to call it a multidimensional sensitivity analysis. Note that in light of the discussion above, attention is restricted to scenarios with *α_u_* < 0, and *α_xu_*, *α_yu_* both non-zero. By focussing on situations with *α_xu_* = ±1 and *α_yu_* = ±1, we consider conditional odds ratios of 2.72 and 1/2.72 = 0.37 between *U* and *X* given *Y*, and between *U* and *Y* given *X*. The choices of *α_u_* = −2 and *α_u_* = −1 correspond to the prevalence of *U* = 1 amongst unexposed controls being 12% and 27% respectively.

One way to summarize the findings from Table 2, or the findings from a more exhaustive TSA involving a wider variety of settings for bias parameters, is via ranges. For instance, across the eight scenarios considered in Table 2, the point estimate for the (*X. Y*|*U*) odds ratio ranges from 2.16 to 3.34. A challenge, however, in interpreting this range is that it ignores sampling variability. Alternately, one might form an overall interval from the constituent 95% confidence interval endpoints by taking the ‘lowest of the low’ and the ‘highest of the high,’ *i.e.*, (1.31, 5.52) in the present situation. Clearly this strategy is highly conservative, since, roughly put, each endpoint corresponds to a pairing of the most extreme scenario with the most extreme random sampling error.

An alternative strategy is a *probabilistic sensitivity analysis* based on sampling scenarios for how *U* relates to the other variables from a distribution over such scenarios. The underlying notion is that we wish to use this distribution over scenarios to induce a distribution of plausible values over the target parameter of interest. In the present context we could achieve this by repeating the following three steps many times.

1. Sample a value for each of (*α_u_*_,_ *α_xu_*, *α_yu_*) from a chosen distribution over scenarios.
2. Based on the selected scenario, sample a value of *U* for each study subject.
3. Based on the resulting set of ‘completed’ data (actual values of *X* and *Y* augmented with sampled values of *U*), sample a value of *β_xy_* which is consistent with these data.

The ensemble of generated *β_xy_* values then is taken to represent uncertainty about this target arising from uncertainty about which scenario applies (Step 1), plus uncertainty about the actual values of the unobserved variable for the study subjects given the scenario (Step 2), plus the usual statistical uncertainty in estimating population parameters from a sample were all the variables observed (Step 3). Following [3], we refer to this procedure as *Monte Carlo Sensitivity Analysis* (MCSA).

While Step 1 simply involves computer-implemented random sampling from the chosen distribution over scenarios, and Step 2 involves such sampling dictated by Equation (1), Step 3 requires more comment. We instantiate this step by generating *β* from a normal distribution centred at the maximum likelihood estimate of *β* based on the augmented data, with a variance matrix taken to be the estimated variance matrix of the MLE based on the curvature of the log-likelihood at the MLE. This is elaborated upon in the Appendix.

We illustrate this method by assigning the following distribution to the bias parameters, under which *α_u_*, *α_xu_*, and *α_yu_* are taken to be independent of one another. A uniform distribution between −2 and −1 is assigned to *α_u_*, while both *α_xu_* and *α_yu_* are given uniform distributions between −1 and 1. Thus the scenarios considered in Table 2 form the ‘corners’ of the distribution chosen. The induced ensemble of 50, 000 exp(*β_xy_*) values from the MCSA has 2.5*^th^* and 97.5*^th^* percentiles of 1.64 and 4.59. This range of plausible values is less conservative (*i.e.*, narrower) than that obtained as the amalgamation of the 95% confidence intervals under the eight scenarios in Table 2.

The use of uniform distributions over bias parameters lends itself to comparison with the Table 2 approach of looking at all possible combinations of two different values for each bias parameter. It is not very defensible in real problems, however, to argue that all values inside an interval are equally plausible, while all values outside are impossible. Thus we consider replacing the uniform distributions on *α_u_*, *α_xu_*, *α_yu_* with normal distributions that tail off smoothly. To illustrate, we select normal distributions having the same 95% central intervals as the uniform distributions considered. For instance, the uniform distributions between −1 and 1 used for *α_xu_* and *α_yu_* are replaced with normal distributions having mean zero and standard deviation 0.95/1.96 ≈ 0.485. This change in distribution over scenarios actually has virtually no impact on the central 95% interval for the induced distribution over the target odds-ratio, *i.e.*, the endpoints we compute are within Monte Carlo simulation error of the interval (1.64, 4.59) reported above.

As a more formal step beyond MCSA, we consider *Bayesian sensitivity analysis* (BSA). Formally this involves specifying a prior distribution over *all* parameters (not just the bias parameters), which, upon combination with the observed data, yields the *posterior distribution* over the parameters. This distribution over the target parameter is taken as the basis for inference. In many problems we can intuit that MCSA and BSA will yield similar results. In fact, as outlined in the Appendix, we can even use the MCSA results as a stepping stone to obtaining BSA results. More formally, importance sampling can be used to *weight* the MCSA sample of parameter values as a route to obtaining the BSA distribution. In the present situation this weighting moves the 95% central interval away from the null slightly, *i.e.*, the BSA interval on exp(*β_xy_*) arising from the uniform prior distribution is (1.69, 4.79). Again replacing the uniform prior distributions with ‘matching’ normal distributions has a negligible impact on the BSA interval. Some more specialized applications of Bayesian techniques for unobserved confounding can be found in [17, 18].

## 3. Example: Selection Bias in Case-Control Studies

In case-control studies, selection bias can result from the manner in which controls are sampled. Conceptually one can regard the case-control design either as comparing exposure patterns amongst those diseased to exposure patterns amongst the general population, or as comparing exposure patterns amongst those diseased to exposure patterns amongst the disease-free portion of the population. For most applications the disease is very rare in the study population, in which case the distinction between these two formulations becomes unimportant. What matters is that the scheme to select cases yields an exposure prevalence matching the target population.

In the CPC example, the cases were hospital patients with pancreatic cancer. The controls were sampled from patients who were hospitalized by the same attending physicians who had hospitalized the cases. MacMahon *et. al.* [14] argued that the coffee consumption in the controls was a good approximation of coffee consumption in the general population without pancreatic cancer. The problem is that by virtue of being hospitalized by a physician, the controls may have had illnesses that led them to reduce their coffee consumption prior to enrollment in the study. Thus their exposure distribution would be abnormally low compared to the general population. Unmeasured illness in the controls may have induced an inverse association between the exposure and the probability of being selected into the study as a control. Because the exposure and disease both affect the probability of selection, the significant odds ratio discovered in [14] could be a spurious artifact of bias.

In fact, the possibility of selection bias was acknowledged in [14]. However the authors argued that any impact of the controls’ health status on coffee consumption would be negligible compared to the differences observed between cases and controls. They concluded that *“It is inconceivable that this bias would account for the total difference between cases and controls, but it is possible that the risk may be either overestimated or underestimated on this account.”*

The study generated great controversy because coffee drinking is so common. In later years, researchers tried unsuccessfully to replicate the study findings, and the relationship between coffee drinking and pancreatic cancer has been largely refuted [19]. Interestingly, MacMahon *et. al.* [14] did not attempt to study the robustness of their results to quantitative assumptions about selection bias.

We now use this example to illustrate how to adjust for selection bias. In case-control studies, selection bias occurs when the exposure affects the probability of participating in the study. This dependence has the effect of censoring the exposure distribution so that the odds ratio is no longer unbiased. More generally, selection bias results from conditioning on a variable that is affected by both the exposure and disease. It can also emerge in prospective studies, in which case it is called informative censoring. See Hernán [10] for a review.

As before, let *X* and *Y* model exposure and disease status. We introduce an additional variable *S*, which is an indicator for whether an individual is selected into the study. We have *S* = 1 if the subject is selected and *S* = 0 otherwise. Thus the complete data are replicates of (*S*, *X*, *Y*), with probability model *p*(*S*|*X*, *Y*)*p*(*Y*, *X*), but we only observe data when *S* = 1.

A simple formula for adjusting for selection bias is given by Greenland [13] and Lash *et. al.* [8]. We have

(3) $$OR_{\text{XY}}=OR_{\text{XY}|S=1}\times B,$$

where *OR_XY_*_|_*_S_*_=1_ = Odds(*Y* = 1|*X* = 1, *S* = 1)*=*Odds(*Y* = 1|*X* = 0, *S* = 1) is the unadjusted odds ratio for the observed association between *X* and *Y*, while *OR_XY_* = Odds(*Y* = 1|*X* = 1)/Odds(*Y* = 1|*X* = 0) is the target association uninfluenced by selection. In equation (3), the quantity *B* is a ‘bias factor,’ given as

$$B=\frac{\text{Pr}(S=1|Y=1,X=1)}{\text{Pr}(S=1|Y=0,X=1)}/\frac{\text{Pr}(S=1|Y=1,X=0)}{\text{Pr}(S=1|Y=0,X=0)},$$

which is computed from the selection probabilities *P*(*S* = 1|*X*, *Y*) over levels of *X* and *Y*.

In the pancreatic cancer example, the crude odds ratio *OR_XY|S_*_=1_ computed from Table 1 is 2.75. Consequently, in order to use equation (3) to adjust for selection bias, it suffices to obtain reasonable prior guesses for the selection probabilities *P*(*S* = 1|*X*, *Y*). Following Greenland [6], we can using a logistic regression model

$$\text{logit}\text{Pr}(S=1|X,Y)=\theta_{0}+\theta_{x}X+\theta_{y}Y+\theta_{\text{xy}}\text{XY}.$$

In this case, the odds ratios exp(*θ_x_*) and exp(*θ_y_*) govern the main effects of *X* and *Y* on the probability of selection. The intercept *θ*_0_ determines the probability of selection among the controls with zero exposure, and *θ_xy_* is a potential interaction term. Thus the collection (*θ*_0_, *θ_x_*, *θ_y_*, *θ_xy_*) constitute the bias parameters that are needed to characterize the selection process and adjust for selection bias.

To illustrate bias adjustment in action, Table 3 gives values of *OR_XY_* in the cancer data as we toy with the bias parameters *θ_x_*, *θ_y_* and *θ*_0_. To generate the table, we lock the parameter *θ_xy_* = 0, and then compute the bias factor *B* and commensurate bias-adjusted *OR_XY_* over a range of values for (*θ*_0_, *θ_x_*, *θ_y_*). By setting *θ_xy_* = 0 we assume that the exposure and disease do not interact in determining probability of selection. This is a fairly realistic assumption in the pancreatic data example. MacMahon *et. al.* [14] speculate that unmeasured chronic disease could induce an inverse association between coffee consumption and selection probability, and furthermore that this mechanism would operate similarly in both cases and controls.

As was the case with unmeasured confounding, the bias parameters *θ_x_* and *θ_y_* enjoy a symmetry property in the sense that for a given *θ*_0_ we see distinct combinations of (*θ_x_*, *θ_y_*) generating the same bias factor *B*. We can eliminate this redundancy by limiting our sensitivity analysis to the case where *θ_y_* > 0, which assumes that the cases have a higher probability of selection than the controls. Of course, this is implicit for case-control sampling, so forcing *θ_y_* > 0 is natural and inconsequential in the sensitivity analysis.

Table 3 displays the sensitivity of the odds ratio for different assumptions about the manner in which the exposure and outcome affect the probability of selection. We let *θ_x_* and *θ_y_* be as large as 1 in magnitude, which corresponds to an odds ratio of exp(−1) = 0.37 or exp(1) = 2.71. If *θ_x_* = −1 for example, then this implies that coffee drinking reduces one’s odds of participating in the study by a factor of 0.37. We set *θ*_0_ equal to −3 or −5 to reflect that non-cases in the population have very low probability of being included as controls in the study, with probabilities expit(−3) = 5% or expit(−5) < 1%.

In Table 3, we see that as we vary the bias parameters the odds ratio shifts toward or away from the null value of 1. Standard errors and commensurate 95% confidence intervals are calculated from the sandwich variance estimate of weighted logistic regression of *Y* on *X*, as described below. Setting either *θ_x_* or *θ_y_* equal to zero implies no bias and this is manifested as an odds ratios equal to 2.75, matching the odds when ignoring selection bias. This is also apparent from equation (3). In the absence of interaction between *X* and *Y* on the probability of selection, we see that *B* = 1 if either *θ_x_* = 0 or *θ_y_* = 0.

The significant association between coffee drinking and cancer is very robust to different assumptions about selection bias. For example, the bias parameter combination (*θ*_0_, *θ_y_*, *θ_x_*) = (−5, 1, −1) is perhaps the most realistic, and yields an odds ratio of 2.73, which is only negligibly different from 2.75. This sensitivity analysis posits that cases have 2.73 *greater* odds of participating than controls, irrespective of exposure status, and furthermore, that coffee drinkers have 2.73 times *lesser* odds of participating, irrespective of disease status. Thus the bias-corrected odds ratio is attenuated slightly towards the null value of 1. The robustness of the odds ratio to bias is somewhat surprising and lends support to the original claim in [14] that selection bias alone could not explain the results.

Taking the analysis a step further, we can conduct MCSA where we assign probability distributions to the bias parameters (*θ*_0_, *θ_x_*, *θ_y_*, *θ_xy_*). We model our prior beliefs about the magnitude of bias and then incorporate them into the analysis. We assume that (*θ*_0_, *θ_x_*, *θ_y_*, *θ_xy_*) are *a priori* independent, with *θ_xy_* = 0 and assume that *θ*_0_ ∼ *N*(−5, 1^2^), meaning that we believe that the probability of selection for unexposed controls lies between 0.1% and 5% with probability 95%. We assign *θ_y_* ∼ *N*(8, 1.5^2^) to indicate that cases have roughly +8 greater log-odds of being selected in the study. This seems reasonable because presumably nearly all pancreatic cancer cases are recruited into the study, whereas disease free individuals constitute the bulk of the study population. Finally we set *θ_x_* ∼ *N*(−0.3, 0.1^2^). This last prior is determined based on the 2 × 2 table presented in Table 4 of Rosenberg *et. al.* [20], who MacMahon *et. al.* [14] cited in their effort to defend their results from controversy about selection bias. Particularly, MacMahon *et. al.* argued that coffee drinking is only modestly associated with having a chronic illness, and therefore the probability of being recruited as a control into the study. Thus we can now examine how formalized assumptions about bias play out in a probabilistic sensitivity analysis.

Paralleling the discussion of MCSA in Section 2, we repeatedly implement the following three step procedure described by Greenland [6].

1. Draw a value of (*θ*_0_, *θ_x_*, *θ_y_*, *θ_xy_*) from the prior distribution.
2. Compute the inverse probabilities of selection, {expit(*θ* + *θ_x_X* + *θ_y_Y*)}^−1^, as weights for fitting a weighted logistic regression of *Y* on *X*.
3. Based on the fit, use the estimated *X* coefficient and its standard error as the mean and standard deviation in drawing a single normally-distributed value for log *OR_XY_*.

By repeating the steps a large number of times, an ensemble of values for log *OR_XY_* is generated. Using the prior distributions outlined above we obtain the mean and standard deviation of the ensemble as 0.75 and 0.28, with 95% central interval of (0.25, 1.30). Note that the standard deviation of 0.28 is only modestly larger than the standard error of the crude log odds ratio, which is 0.26. Correspondingly, the 95% central interval based on MCSA (again on the log odds ratio scale) is only 7.7% wider than the conventional interval ignoring selection bias. Upon exponentiating, the MCSA estimate of *OR_XY_* is 2.12, with 95% interval estimate (1.28, 3.66).

Based on these results we arrive at several conclusions. Plausible priors on the bias parameters (*θ*_0_, *θ_x_*, *θ_y_*, *θ_xy_*) do indeed shift the odds ratio for the coffee and pancreatic cancer association toward the null (from 2.75 to 2.12). However, the statistical significance of the result is unaffected. While this bias is important it does not appear to be the major source on uncertainty in the study. Thus the coffee cancer relationship is indeed insensitive to realistic assumptions about selection bias, as MacMahon *et. al.* [14] originally argued.

It is difficult to know how to interpret these results. Selection bias alone does not appear to be driving the association in Table 1. Since later research was unable to replicate the coffee-cancer association it suggests we look elsewhere for explanations. One possibility is that the association arose through multiple comparisons and poor type I error control. Another explanation is that there may have been several biases at play at the same time. For instance, coffee consumption may have been measured with error in cases and controls, and relevant confounding variables may not have been identified.

We conclude with a discussion of a full Bayesian treatment of selection bias. Interestingly, unlike other biases, selection bias adjustment does not have a natural Bayesian interpretation. The reason is that the bias adjustment formula in Equation (3) is an inverse probability weighting estimator [10]. The formula is derived by reweighing the exposure distribution using the probabilities of selection. This estimator does not arise through Bayes theorem because there is no likelihood function, which is essential in a Bayesian analysis. For 2 × 2 tables, a Bayesian approach is described by Greenland [6]. Other recent work in this area is given by Geneletti *et. al.* [21].

## 4. Exposure Misclassification

In the coffee and pancreatic cancer example, exposure status is obtained by asking study participants about coffee consumption via a questionnaire. Given that exposure is defined in this study as any non-zero level of daily consumption, one might anticipate that the answers provided by participants are quite accurate in this setting. The situation would be muddied, however, for participants whose consumption pattern may have changed over time. More generally, concern about accurate measurement or classification of exposure abounds in epidemiology. Typically this falls under the heading of exposure measurement error when the exposure variable is continuous, and exposure misclassification when the exposure variable is categorical (with absent/present being an important special case).

To give an illustration of how we might conduct an analysis which acknowledges exposure misclassification, say the relationship of interest is

(4) $$\text{logitPr}(Y=1|X)=\beta_{y}+\beta_{xy}X.$$

Thus we focus on a situation where adjustment for confounding is not attempted. Rather, a challenge arises in that the observed data are (*W*, *Y*) rather than (*X*, *Y*). That is, our available binary exposure measurement *W* is imperfect, and will differ from the true binary exposure *X* for some subjects. The rate or extent of corruption can be characterized by sensitivity and specificity, *i.e.*, *SN* = *Pr*(*W* = 1|*X* = 1), *SP* = *Pr*(*W* = 0|*X* = 0).

To focus on a simple case for illustration, we consider *nondifferential* misclassification, whereby the proclivity for exposure misclassification is independent of outcome. This can be expressed as conditional independence of *W* and *Y* given *X*, allowing us to extend (4) to the form

(5) $$\text{logitPr}(Y=1|X,W)=\beta_{y}+\beta_{xy}X,$$

whilst also postulating that

(6) $$\text{logitPr}(W=1|X,Y)=\alpha_{w}+\alpha_{xw}X.$$

Thus (*α_w_*, *α_xw_*) can be regarded as bias parameters, interpretable via *SN* = expit(*α_w_* + *α_xw_*), and *SP* = 1 − expit(*α_w_*) = expit(−*α_w_*). We emphasize that focus on the nondifferential case is for ease of exposition, and we fully recognize that *differential* misclassification (*SN* and *SP* vary with *Y*) can easily arise in many situations. In fact, settings where a binary exposure is defined by thresholding a continuous exposure variable will typically lead to differential misclassification. More precisely, this happens if an error-prone surrogate for the continuous exposure is thresholded to create an error-prone binary exposure, when the risk of disease varies smoothly with the continuous exposure [22, 23]. Another way that differential misclassification occurs is via differential recall of exposure via questionnaire, where a subject’s disease status may exert a subtle influence on his/her recollection concerning exposure.

Note that the present framework is somewhat parallel to our treatment of an unobserved confounder in Section 2, with two regression relationships at play. That is, (5), like (2) before, governs the relationship of scientific interest, while (6), like (1) before, governs the link between observed and unobserved variables. Moreover, just as before, (5) and (6) can be subsumed by a Poisson model for the counts of subjects stratified by (*X*, *Y*, *W*).

Despite the appealing similarity, it is more challenging to provide some form of probabilistic sensitivity analysis in the present setting than in the Section 2 setting. The challenge arises via the fact that the distribution of the unobserved variable *X* given observed variables (*Y*, *W*) depends not just on bias parameters *α* = (*α_w_*, *α_xw_*), but also on *β_xy_* as well. Thus the earlier strategy of (i), simulating bias parameters *α* from their prior, (ii), simulating commensurate complete data, and (iii), simulating *β* given complete data, is no longer applicable. Rather, we turn to some heavier machinery of Bayesian computation, namely Markov chain Monte Carlo algorithms. While a review of these algorithms is beyond the scope of this paper, we can call upon the WinBUGS software [24], a general-purpose software package for computing posterior distributions based on inputted model, prior, and data specifications. Some details on handling the present problem via WinBUGS appear in the Appendix.

With case-control data and nondifferential misclassification, we can focus on the four parameter model studied in detail in Gustafson, Le and Saskin [25], rather than the more general Poisson model for cell counts. The four unknown parameters are exposure prevalences amongst controls and cases, *Pr*(*X* = 1|*Y* = *y*) for *y* = 0, 1, and sensitivity and specificity. A simple prior distribution treats these four parameters as independent, with uniform priors (between zero and one) for each exposure prevalence, and Beta priors (also between zero and one) describing plausible values for *SN* and *SP*. As an example, say that both *SN* and *SP* are ascribed Beta(13.5,1.5) prior distributions. This distribution has mean 0.9 and mode 0.893, and puts only 5% probability on values below 0.75. Applied to the CPC data via WinBUGS, the resulting posterior distribution on the target parameter is extremely wide, with 95% central interval (1.99, 75.1) on the odds-ratio exp(*β_xy_*).

To explore this further, the 95% central interval above translates to (0.67, 4.44) on the log odds-ratio, *i.e.*, the interval for *β_xy_*. Moreover, the posterior distribution is somewhat asymmetric, with a mean of 1.79. In comparison, the 95% confidence interval assuming perfect exposure classification is (0.51, 1.52), centered around the point estimate of 1.01 (again on the log odds-ratio scale). Thus, relative to pretending that the exposure classification is perfect, the point estimate is pushed away from the null considerably, and the interval estimate is widened very considerably. To large extent these two changes ‘cancel out’ in terms of the lower bound of the interval estimate only moving modestly away from the null value of zero.

In fact, even in the simpler setting of known (*SN*, *SP*) values one sees that adjustment for nondifferential misclassification pushes estimates away from the null, since the adjustment compensates for the necessarily weaker association between *W* and *Y* compared to that between *X* and *Y*. It should be noted, however, that often such adjustment does not strengthen evidence about the direction of the effect [26, 27]. Also, the widening of the interval arises via two sources. First, even if (*SN*, *SP*) were known, adjustment would acknowledge that (*W*, *Y*) data are less informative than (*X*, *Y*) data when the (*X*, *Y*) relationship is of interest. Second, the interval also acknowledges the uncertainty about (*SN*, *SP*) values, as encoded in the prior distribution.

**Figure 1.**
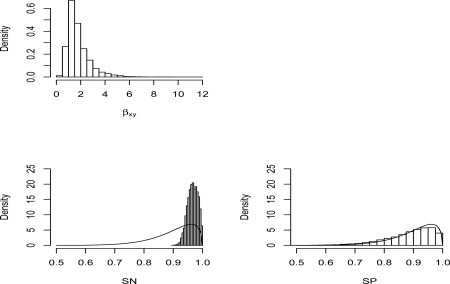
Posterior distributions of *β_xy_*, *SN*, *SP* after acknowledging nondifferential misclassification. The prior densities of *SN* and *SP* are indicated via smooth density curves.

A particular point of interest in this example is comparison of the prior and posterior distributions on the bias parameters, *SN* and *SP*. These are displayed in Figure 1, along with the posterior distributions on the target parameter of interest *β_xy_*. We are accustomed to sensitivity analysis problems where the bias parameters are not informed by the observed data, and we see this to be the situation for *SP*. Curiously, however, the posterior distribution of *SN* is much more concentrated than the prior distribution. There are several points to be made about this. First, there is an intuitive reason why it happens. A classification as exposed can result from correct assessment of an exposed subject or from incorrect assessment of an unexposed subject. Thus

(7) $$\begin{array}{l} \text{Pr}(W=1|Y=y)=\text{Pr}(X=1|Y=y)\text{SN}+\\ \;\;\;\;\;\;\;\;\;\;\;\;\;\;\;\;\;\;\;\;\;\;\;\;\;\;\;\;\;\;\;\;\;\;\;\;\;\;\;\;\;\;\;\{1-\text{Pr}(X=1|Y=y)\}(1-\text{SP}), \end{array}$$

and consequently *Pr*(*W* = 1|*Y* = *y*) must lie between 1 − *SP* and *SN*. The present data provide very strong evidence that *Pr*(*W* = 1|*Y* = *y*) is large, for both *y* = 0 and *y* = 1. This then serves to rule out some lower values of *SN*, at least probabilistically. In fact, the present data are unusual for an epidemiologic setting. It is much more common to encounter low-prevalence exposures. In such settings lower values of *SP* may be discredited, by the same sort of logic described above.

As a second point, the fact that substantial prior-to-posterior updating of a bias parameter can be seen, at least for some datasets, speaks against the applicability of TSA or MCSA in such settings. With TSA or MCSA, their is no attempt to access any information *in the data* about relative plausibilities of values for the bias parameters.

## 5. Discussion

Probabilistic sensitivity analysis is a new suite of methods to help epidemiologists deal with bias in observational studies. On the one hand, such methods permit shifting odds-ratios away from or toward the null value, based on beliefs about biasing mechanisms. Moreover, they also give more plausible quantifications of uncertainty, as they incorporate uncertainties about the magnitude and direction of bias. For example, in the CPC data example, the actual amount of selection bias is debatable. It could be large or small depending on assumptions about the probabilities of selection. Prior distributions on bias parameters incorporate this uncertainty into the analysis.

The methods are particularly useful in settings where confidence intervals around effect estimates are small, e.g., in meta-analysis or studies using large population databases. In such settings random sampling variation contributes only a small fraction of the total uncertainty that is at play. Thus probabilistic bias analysis gives a more honest assessment of the major sources of uncertainty.

In the CPC example, we illustrated that the association between coffee drinking and cancer is robust to different assumptions about selection bias. These findings are surprising in light of the fact that the study results were never successfully replicated. One possible interpretation is that there were multiple biases involved in the data collection process. Indeed the simultaneous modeling of *multiple* biases is a further refinement of sensitivity analysis techniques that we have not discussed, but is a major focus in [3, 6]. For example, in addition to selection bias it is possible that coffee consumption was measured with error in cases and controls, and that this may have further exaggerated the association between exposure and disease.

One outstanding question is determining why exactly the odds ratio in Table 1 is so robust to bias. Certainly the sample size and the magnitude of the crude association between *X* and *Y* should play a role. But the situation is more complex in studies with covariate data. In general, there has been little research characterizing the design considerations when one hopes to minimize sensitivity to bias. Some recent work in this area is given by Rosenbaum [28]. One possibility is to make formal use of the notion of ‘prior data’, described by Greenland [29], to help understanding the information content of prior distributions by representing these distributions in the form of ‘extra’ data points.

## Figures and Tables

**Table 1.** Case-control data for coffee drinking and pancreatic cancer.

		Cases	Controls	
Coffee drinking (cups per day)	≥ 1	347	555	902
	0	20	88	108
		367	643	1,010

**Table 2.** Point estimate and 95% confidence interval for target parameter under different values of bias parameters. Recall that under the assumption of no unobserved confounding the estimated odds ratio is 2.75, with a 95% confidence interval of (1.66, 4.55). The method for determining the confidence intervals is described in the Appendix.

*α_U_*	*α_XU_*	*α_YU_*	exp (*β_XY_*)	95% CI	
−2	−1	−1	2.62	1.58	4.34
−2	−1	1	3.06	1.85	5.07
−2	1	−1	3.06	1.85	5.07
−2	1	1	2.27	1.37	3.75
−1	−1	−1	2.47	1.49	4.09
−1	−1	1	3.34	2.02	5.52
−1	1	−1	3.34	2.02	5.52
−1	1	1	2.16	1.31	3.58

**Table 3.** Bias corrected odds ratios *OR_XY_* as a function of (*θ*_0_; *θ_x_*; *θ_y_*) in the case where *θ_xy_* = 0.

*θ*_0_	*θ_y_*	*θ_x_*	*OR_XY_*	95% CI	
−5	0	−1	2.75	1.66	4.55
−5	0	0	2.75	1.66	4.55
−5	0	1	2.75	1.66	4.55
−5	1	−1	2.73	1.65	4.52
−5	1	0	2.75	1.66	4.55
−5	1	1	2.80	1.69	4.64
−3	0	−1	2.75	1.66	4.55
−3	0	0	2.75	1.66	4.55
−3	0	1	2.75	1.66	4.55
−3	1	−1	2.62	1.58	4.34
−3	1	0	2.75	1.66	4.55
−3	1	1	3.06	1.85	5.07

## Appendix

### Further Details for Section 2

In the unobserved confounding setting, let *c_xyu_* be the count of study subjects having *X* = *x*, *Y* = *y*, *U* = *u*. The constituent logistic regression models (1) and (2) can be subsumed by a log-linear model for the cell counts, *i.e.*, a Poisson model with

(8) $$\text{log}E(c_{\text{xyu}})=\beta_{0}+\beta_{x}X+\beta_{y}Y+\beta_{\text{xy}}\text{XY}+\alpha_{u}U+\alpha_{\text{xu}}\text{XU}+\alpha_{\text{yu}}\text{YU}.$$

If (*X*, *Y*, *U*) were observed for all study subjects, so that *c_xyu_* were observed for all cells, then (8) could be fit to the data using any standard software package for fitting log-linear models, resulting in estimates of both sets of parameters, *α* and *β*. As usual, this could be carried out for cohort-study data or case-control data, with the proviso that some parameters are not sensibly estimated from case-control data, e.g., *β_y_* which governs the baseline disease risk in the population.

Turning to the real problem at hand with *U* unobserved, TSA corresponds to fitting (8) with *α* taken as known, but with only partially observed data. In general, fitting generalized linear models with some parameters fixed at ‘known’ values is simply done via standard software. In particular, the terms involving known parameters are specified as an *offset* in the *linear predictor*. The issue of partially observed data, however, requires more attention.

In the present setting, for each level of *x* and *y*, *c_xy•_* = *c_xy_*_0_ + *c_xy_*_1_ is observed, while (*c_xy_*_0_, *c_xy_*_1_) = (*c_xy•_* − *c_xy_*_1_, *c_xy_*_1_) are unobserved. As a general technical point about fitting models involving unobserved data, the *score function* (first derivative of the log-likelihood) arising from the observed data can be expressed as the conditional expectation of the complete-data score function, where this conditional expectation is with respect to the distribution of the complete data given the observed data. In the present situation this yields substantial simplification. Specifically, in light of (1), the distribution of complete data given observed data is characterized by a binomial distribution of *c_xy_*_1_ given *c_xy_*_•_, with expectation *c̄_xy_*_1_(*α*) = *c_xy_*_•_expit(*α_u_* + *α_xu_x* + *α_yu_y*). The facts that (i), this conditional distribution does not depend on the unknown parameters *β*, and (ii), the complete-data score function is linear in the cell counts *c_xyu_*, imply that the observed-data score function can be obtained by pretending that the *c̄_xyu_*(*α*) constitute the complete-data cell counts. Thus we can plug these ‘pseudo-counts’ (which generally won’t be integers) into standard log-linear model software to fit model (8), with *α* known and *β* unknown. Since this induces the software to work with the correct score function, the point and interval estimates returned will be correct. Several points should be made about this scheme to obtain estimates. First, some software packages may produce ‘warnings,’ when asked to fit a Poisson model to responses that are non-integer–this is certainly the case with the **glm()** function in the R environment (www.r-project.org)–but the output will still be as desired. Second, the scheme can in fact be regarded as an application of the Expectation-Maximization (EM) algorithm to the problem. Since the conditional expectation required in the ‘E-step’ does not depend on the unknown parameters *β*, the algorithm converges to the maximum likelihood estimate in a single iteration.

Now we consider the MCSA scheme. We formally denote the missing and observed data as *c_mis_* = (*c*_001_, *c*_011_, *c*_101_, *c*_111_) and *c_obs_* = (*c*_00•_, *c*_01•_, *c*_10•_, *c*_11•_), noting that the complete data *c_com_*, comprised of all *c_xyu_* counts, are determined by (*c_mis_*, *c_obs_*). The MCSA algorithm can be expressed as repeatedly sampling (*α*, *β*, *c_mis_*) from the joint density given by:

(9) $$p_{\text{MCSA}}(\alpha ,\beta ,c_{\text{mis}}|c_{\text{obs}})=p(\alpha )\left\{\prod_{i,j}p(c_{\text{ij}1}|c_{\text{ij}•},\alpha )\right\}p(\beta |c_{\text{com}},\alpha ).$$

Here *p*(*α*) is the selected prior distribution, while *p*(*c_ij_*_1_|*c_ij_*_•_, *α*) is the conditional distribution implied by (1), *i.e.*, the binomial distribution detailed above. The term *p*(*β*|*c_com_*, *α*) denotes the multivariate normal distribution with mean and variance determined from fitting the log-linear model (8) to the complete data with *α* taken as known. Particularly the mean and the precision matrix are taken to be the maximum likelihood estimate of *β* and the second derivative of the log-likelihood evaluated at this estimate, respectively.

In contrast, under a fully Bayesian analysis the posterior density is

(10) $$\begin{array}{l} p_{\text{BSA}}(\alpha ,\beta ,c_{\text{mis}}|c_{\text{obs}})\propto p(\alpha )p(\beta )\prod_{x,y,u}f(c_{\text{xyu}}|\alpha ,\beta )\\ \;\;\;\;\;\;\;\;\;\;\;\;\;\;\;\;\;\;\;\;\;\;\;\;\;\;\;\;\;\;\;\;\;\;\;\;\;\;\;\;\;\;\propto p(\alpha )p(\beta )\prod_{x,y}f(c_{\text{xy}•}-c_{\text{xy}1}|\alpha ,\beta )f(c_{\text{xy}1}|\alpha ,\beta ), \end{array}$$

where *f*(*c_xyu_*|*α*, *β*) is based on the Poisson model (8). Thus (10) arises from a standard amalgamation of a prior distribution on all parameters (*α* and *β* here) with a likelihood function. One can compute the BSA from the MCSA by using *importance sampling* to reweight the MCSA sample. Importance sampling is a standard tool for converting a simple random sample from one distribution into a weighted sample from a different distribution. Specifically, the weight for a particular (*α*, *β*, *c_mis_*) draw is simply proportional to the ratio of the desired density (10) to the sampled density (9). Note that the *p*(*α*) term will cancel in this ratio.

The weighted sample represents the BSA results just as the unweighted sample represents the MCSA results. For instance, the Bayesian 95% interval for the target parameter *β_xy_* could be formed as the 2.5-th and 97.5-th percentiles of the weighted sample (*i.e.*, points with 2.5% of the total weight below/above). Informally, one can plot the weights against the sampled *β_xy_* values to quickly assess whether the BSA result is substantially different from the MCSA result. Note also that plotting the weights against the components of *α* reveals whether the posterior distribution of *α* differs in any substantial way from the prior distribution (since by construction the MCSA involves sampling *α* from its prior distribution). This is a generally useful technique, since in many sensitivity analysis settings one intuits that the data are not informative about the bias parameters, but this cannot be established with mathematical certainty.

The R code we developed to implement the TSA, MCSA, and BSA analyses is posted online (www.stat.ubc.ca/~gustaf). Also, the TSA and MCSA analsyes are readily implemented via The Stata package developed by Orsini *et. al.* [30]. Lash *et. al.* [8] provide SAS code and Excel worksheets implementing various sensitivity analyses: (sites.google.com/site/biasanalysis).

### Further Details for Section 4

For a case-control setting with binary exposure and nondifferential misclassification, the sampling model can be expressed via the numbers of controls and cases classified as exposed, given the total numbers of controls and cases, as being independent Binomial realizations. The ‘success’ probabilities are given by equation (7). WinBUGS code to specify this model along with the prior distributions described in Section 4 is as follows.

model

{

wtot0 ˜ dbin (p0, n0)

wtot1 ˜ dbin (p1, n1)

p0 <– r0★SN + (1–r0)★(1–SP)

p1 <– r1★SN + (1–r1)★(1-SP)

lor <– logit (r1) – logit (r0)

r0 ˜ dunif (0, 1)

r1 ˜ dunif (0, 1)

SN ˜ dbeta (a. sn, b. sn)

SP ˜ dbeta (a. sp, b. sp)

}

This code requires input of the dataset in the form of wtot0 out of n0 controls and wtot1 out of n1 cases having apparent exposure *W* = 1, and the particular specification of a prior specification via hyperparameters a.sn, b.sn, a.sp, b.sp.
